# Mapping Normative Trajectories of Cognitive Function and Its Relation to Psychopathology Symptoms and Genetic Risk in Youth

**DOI:** 10.1016/j.bpsgos.2022.01.007

**Published:** 2022-02-01

**Authors:** Rikka Kjelkenes, Thomas Wolfers, Dag Alnæs, Dennis van der Meer, Mads Lund Pedersen, Andreas Dahl, Irene Voldsbekk, Torgeir Moberget, Christian K. Tamnes, Ole A. Andreassen, Andre F. Marquand, Lars T. Westlye

**Affiliations:** a Department of Psychology, University of Oslo, Oslo, Norway; b KG Jebsen Centre for Neurodevelopmental Disorders, University of Oslo, Oslo, Norway; c Norwegian Centre for Mental Disorders Research, Division of Mental Health and Addiction, University of Oslo, Oslo University Hospital, Oslo, Norway; d Department of Psychiatric Research, Diakonhjemmet Hospital, Oslo, Norway; e Oslo New University College, Oslo, Norway; f Donders Centre for Cognitive Neuroimaging, Donders Institute for Brain, Cognition and Behaviour, Radboud University, Nijmegen, the Netherlands; g Department of Cognitive Neuroscience, Radboud University Medical Centre, Nijmegen, the Netherlands; h School of Mental Health and Neuroscience Faculty of Health, Medicine and Life Sciences, Maastricht University, Maastricht, the Netherlands; i Department of Neuroimaging, Center for Neuroimaging Sciences, Institute of Psychiatry, King’s College London, London, United Kingdom

**Keywords:** Cognition, Development, Genetic risk, Normative modeling, PNC, Psychopathology

## Abstract

**Background:**

Adolescence hosts a sharp increase in the incidence of mental disorders. The prodromal phases are often characterized by cognitive deficits that predate disease onset by several years. Characterization of cognitive performance in relation to normative trajectories may have value for early risk assessment and monitoring.

**Methods:**

Youth aged 8 to 21 years (*N* = 6481) from the Philadelphia Neurodevelopmental Cohort were included. Performance scores from a computerized neurocognitive battery were decomposed using principal component analysis, yielding a general cognitive score. Items reflecting various aspects of psychopathology from self-report questionnaires and collateral caregiver information were decomposed using independent component analysis, providing individual domain scores. Using normative modeling and Bayesian statistics, we estimated normative trajectories of cognitive function and tested for associations between cognitive deviance and psychopathological domain scores. In addition, we tested for associations with polygenic scores for mental and behavioral disorders often involving cognition, including schizophrenia, bipolar disorder, attention-deficit/hyperactivity disorder, and Alzheimer’s disease.

**Results:**

More negative normative cognitive deviations were associated with higher general psychopathology burden and domains reflecting positive and prodromal psychosis, attention problems, norm-violating behavior, and anxiety. In addition, better performance was associated with higher joint burden of depression, suicidal ideation, and negative psychosis symptoms. The analyses revealed no evidence for associations with polygenic scores.

**Conclusions:**

Our results show that cognitive performance is associated with general and specific domains of psychopathology in youth. These findings support the close links between cognition and psychopathology in youth and highlight the potential of normative modeling for early risk assessment.

Adolescence is a period characterized by substantial biological, cognitive, and psychological development. Supporting increasing demands from the social environment toward independence, the period from early childhood to young adulthood hosts a strong refinement of basic cognitive functions such as sensorimotor processes and complex functions such as cognitive control and emotion regulation (1,2). Accompanying and supporting this gradual improvement of cognitive functions, substantial maturational reorganization across brain tissue types and regions has been reported (3,4). Likely partly explained by a combination of concurrent biological changes and increasing environmental and social demands, adolescence and early adulthood are also accompanied by a sharp increase in the incidence rates of many mental disorders (5,6).

Converging behavioral, neural, and genetic evidence supports a neurodevelopmental component in the etiology of several mental disorders (7, 8, 9). Cognitive deficits are abundant in most mental disorders; however, substantial heterogeneity in cognitive performance has been reported among patients with disorders such as schizophrenia, bipolar disorder, and attention-deficit/hyperactivity disorder (ADHD) (10, 11, 12, 13). Subtle cognitive impairments are often present already in the prodromal phase of mental disorders and are likely to predate the onset of clinical symptoms for months and even years (14). While Alzheimer’s disease (AD) is associated with later life stages, studies have found that disease processes and subtle cognitive dysfunctions may emerge several years and even decades before clinical onset (15), which may point to a neurodevelopmental origin even in late-life cognitive disorders. Previous studies on youths with high polygenic risk for AD have revealed mixed findings, some indicating possible early cognitive alterations (16) and others suggesting no associations between polygenic scores and cognitive performance during childhood and adolescence (17).

ADHD, schizophrenia, bipolar disorder, and AD are all highly heritable disorders (13,18, 19, 20). While parts of the heritability are likely attributed to rare sequence variants with large effects, well-powered genome-wide association studies from the last decade indicate that a large fraction of the heritability of most mental disorders is accounted for by the joint effects of numerous common genetic variants, each with low penetrance (21, 22, 23). Based on this concept of distributed genetic associations, polygenic scores aggregate the genetic load across the genome based on individual-level genotype information (24). This method can afford accurate descriptions of polygenic risk, which, along with charting of individual variation in cognitive development and performance preceding the clinical phase, can be vital for detection of deviations related to risk of psychopathology (25). By mapping individual differences, normative modeling offers a conceptual and statistical framework for quantifying the normative range and individual deviations in the trajectories of biological and other relevant clinical features (26).

To examine patterns and variation of general cognitive functioning across age as an early indicator of mental disorder risk, we applied normative modeling on cognitive test data obtained from 6481 children and adolescents aged 8 to 22 years. Principal component analysis (PCA) was performed on the cognitive test data, and the first principal component was used as a generalized measure of cognition. We used normative modeling to probe each individual’s performance in relation to the estimated trajectories, resulting in an individual overall cognitive performance deviance score. In line with the aims of the National Institute of Mental Health Research Domain Criteria project of mental disorders (27), we decomposed self-reported questionnaire items and collateral information reflecting measures of psychopathology using independent component analysis (ICA) into data-driven and independent patterns of clinical symptom features (28). For the subset of participants with European ancestry and available cognitive, clinical, and genetic data (*n* = 3175), we tested for associations between individual deviance scores and polygenic scores for various mental and cognitive disorders, including schizophrenia, bipolar disorder, ADHD, and AD. Adopting a Bayesian regression framework, we hypothesized that deviating more negatively from the norm on cognition would be associated with higher scores in the selected clinical domains and greater polygenic scores for schizophrenia, bipolar disorder, ADHD, and AD.

## Methods and Materials

### Sample Description

We included data from the Philadelphia Neurodevelopmental Cohort, comprising >9000 participants aged 8 to 21 years (mean = 13.6, SD = 3.6) (29,30). Available information includes medical history; genetic, clinical, and cognitive data; and for a subset, neuroimaging. Details about recruitment procedures, sample characteristics, and clinical, cognitive, and imaging procedures have previously been described (30). Complete cognitive data were available from 6481 participants (3377 females); participants with severe medical conditions (*n* = 44) or missing clinical data (*n* = 4) were excluded. Genetic data were available from 4450 White Europeans; from these, 3175 (*n* = 1581 females, age = 8−21 years) participants also had cognitive and clinical data available (see Table S1 for further descriptions of the two samples).

### Neurocognitive Test Battery

Participants completed a computerized test battery comprising 14 tests assessing executive functions, episodic memory, complex cognition, social cognition, and sensorimotor speed (31). Accuracy and response times were available for all tests. In total, we included 16 performance scores from 14 tests (see Figure S1 for more details). In addition, the Wide Range Achievement Test was administered to provide an estimate of general intellectual abilities. The percentage of missing values for the cognitive items ranged from 0.01% to 2.5%. For participants missing 1 to 5 test scores (*n* = 164), mean imputation was used. Participants missing >5 values were excluded (*n* = 4).

**Figure 1:**
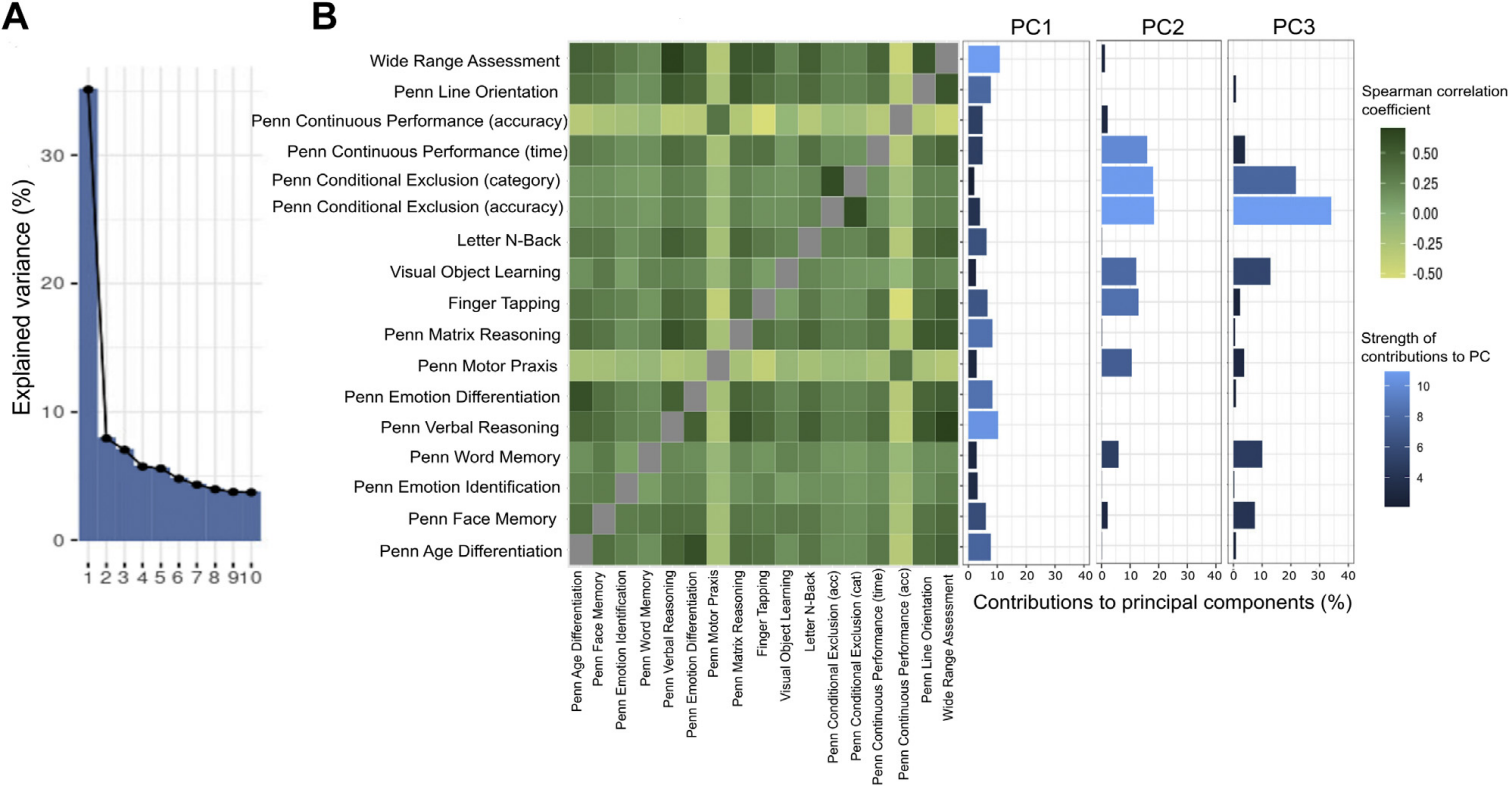
Principal component (PC) analysis output and correlation matrix. **(A)** Scree plot showing the percentage of explained variance of the PCs from the PC analysis. **(B)** Spearman cross-correlation matrix showing the correlations between the different cognitive tests, and a bar plot showing the contributions of each cognitive test to the PCs.

To obtain a data-driven measure of general cognitive function, we performed PCA and extracted the first component as a measure of general cognitive function (Figure 1). This component explained 35% of the variance, with the highest contributions from the Wide Range Achievement Test (10.9%) and the Penn Verbal Reasoning Test (10.4%). The contributions to the other components can be seen in Table S2.

### Normative Modeling

**Figure 2:**
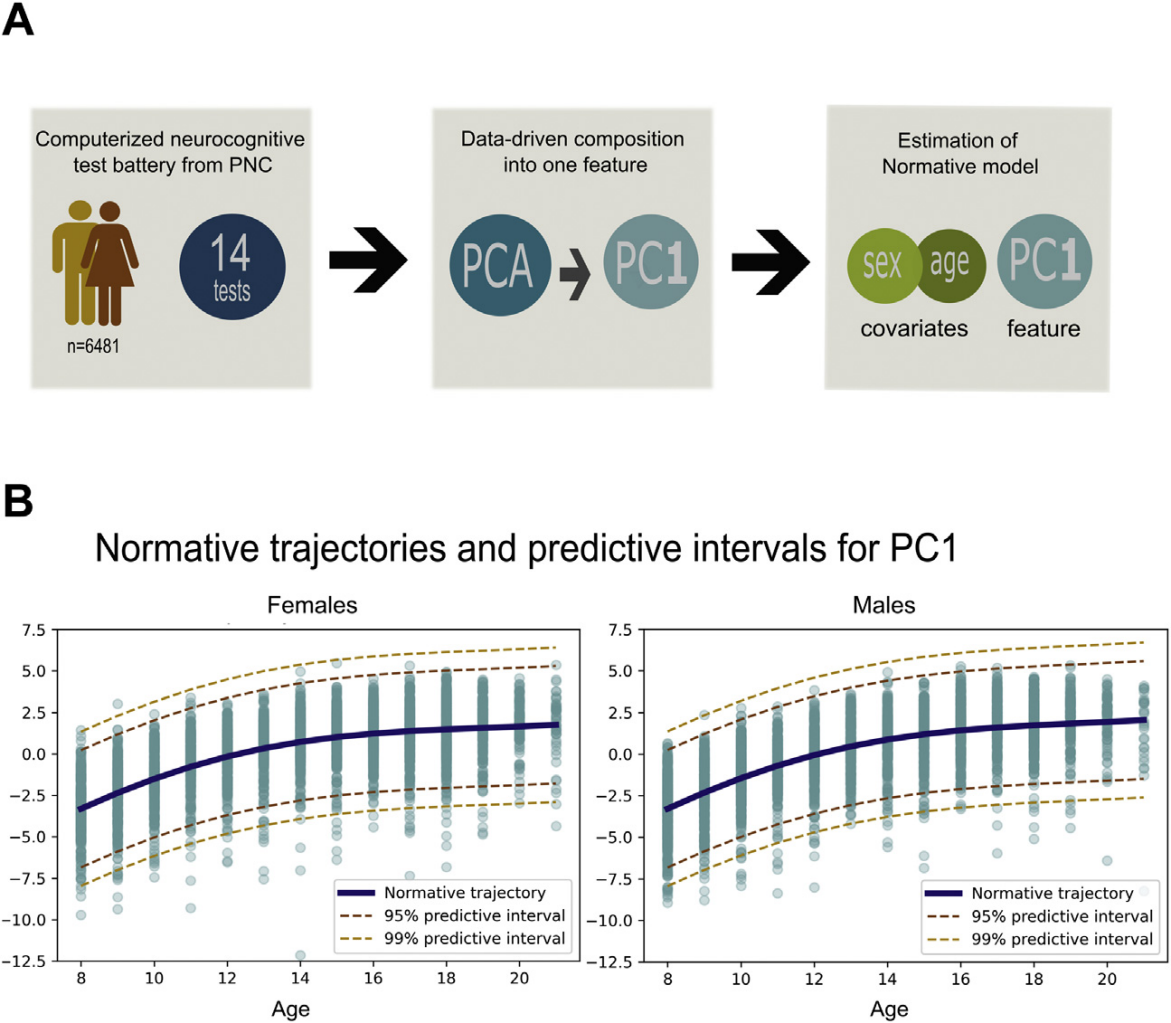
Workflow and visualization of the normative model. **(A)** Overview of the statistical procedure for building the normative models. **(B)** Visualization of the normative trajectories for cognition with predictive intervals and participants’ performance scores plotted on. Here, values have been inverted for visualization purposes. PC, principal component; PCA, principal component analysis; PNC, Philadelphia Neurodevelopmental Cohort.

Figure 2 summarizes the analysis workflow. We used the normative modeling framework to predict the first cognition-based principal component from the covariates sex and age under 10-fold cross-validation with the goal to detect individual-level deviations from the estimated norm. The Gaussian process regression predicted 45.6% of the variance of the observed cognitive performance out of sample. This shows that the PCA component was suited for growth charting because it was sufficiently linked to sex and age (mean standardized log loss = −0.304, root mean squared error between true/predicted responses = 1.803, standardized mean squared error = 0.544).

Thus, our results show an improvement in cognitive performance with increasing age for both males and females. While an association with age is essential for growth charting, the Gaussian process regression provides a consistent measure of predictive confidence in addition to point estimates (32). Using this estimate, we could quantify the deviation of each participant from the predicted mean in terms of an individual *z* score. This measure reflects the difference between the predicted cognitive test score and the observed cognitive score normalized by the estimated uncertainty of the prediction (33). Thus, using the normative modeling framework, we were able to estimate a score that reflected the deviation of a participant at the level of the individual.

### Psychopathology Domains

All participants completed a computerized, structured interview (GOASSESSS) to assess major domains of psychopathology. This included measures of anxiety, mood, behavioral, eating, and psychosis spectrum disorders. In addition, collateral informants were used for participants aged 18 or younger (34). Based on previous work (28), we included 129 items (Table S3) capturing various psychopathological domains from all participants (*N* = 6481). Follow-up and conditional items were not included. The percentage of missing values for the chosen items were between 0% and 7%. For the participants with one or more missing items (*n* = 1627), except for 2 participants who had no clinical data available, missing values were replaced by the nearest neighbor value based on Euclidean distance (28). Based on previous work (28), all available clinical item scores were submitted to ICA using Icasso (MATLAB, version 1.21; The MathWorks, Inc.) (35) to capture latent overlapping categories underlying the psychopathological measures. The items were then decomposed into seven ICs. The resulting ICs represented attention problems (IC1); anxiety (IC2); norm-violating behavior (IC3); positive and prodromal psychosis symptoms (IC4); depression, suicide, and psychosis negative symptoms (IC5); mania (IC6); and obsessive-compulsive symptoms (IC7) (28). In addition, the mean weights across these components were used as a proxy for general psychopathology. Distributions for the included psychopathology domains can be seen in Figure S4.

### Genetic Data

Genotyping was performed by the Center for Applied Genomics at the Children’s Hospital of Philadelphia. The DNA samples from the Philadelphia Neurodevelopmental Cohort were genotyped in different batches using Illumina OmniExpress (*n* = 1657), Illumina Human-610 Quad (*n* = 3807), Illumina HumanHap-550-v1 (*n* = 556), Illumina HumanHap-550-v3 (*n* = 1914), Affymetrix Genome-Wide Human SNP Array 6.0 (*n* = 66), or Affymetrix Axiom (*n* = 722) (hereafter, Omni, Quad, 550-v1, 550-v3, Affy60, and Axiom, respectively). From those datasets, only participants of European ancestry were included in these analyses. Multidimensional scaling and genotype imputation were performed using previously described procedures (36).

Using PRSice version 2 (37) and summary statistics obtained from earlier genome-wide association studies, we computed polygenic scores for schizophrenia (21), bipolar disorder (38), AD (39), and ADHD (22). Polygenic scores were calculated for participants with White European descent (*n* = 4450) for 6002 initial *p*-value thresholds, ranging from 5 × 10^−8^ to 0.5. To avoid setting a random threshold, for each phenotype, we decomposed the full set of polygenic scores into a reduced set of orthogonal components using PCA (40). For all phenotypes, the first components explained between 80% and 85% of the variance and were used in further analyses.

### Statistical Analysis

All statistical analyses were carried out using R, version 3.6.0 (http://www.r-project.org/) (R Core Team, 2012) and python version 3.0 (https://www.python.org/). We used a Bayesian regression approach using the brms (41,42) package in R (R Core Team, 2012) to examine linear associations between deviations from the normative trajectory and either clinical scores or polygenic scores. The deviance scores from the normative cognitive trajectory (i.e., subject-level *z*-statistics) were included as the dependent variable, and age, sex, and either clinical scores or polygenic scores were entered as independent variables. To prevent false positives and to regularize the estimated associations, we used a prior strongly centered around zero (mean = 0, SD = 0.5) for all coefficients. All variables were standardized prior to analysis. We calculated Bayes factors (BFs) using the Savage-Dickey density ratio method (43). BF reflects the strength of evidence in favor of the null hypothesis or the alternative hypothesis. BF = 1 can be interpreted as evidence pulling in either direction. The following values can be interpreted as weight toward the alternative hypothesis with the following strengths: 0.3 to 1 (anecdotal), 0.1 to 0.3 (moderate), 0.03 to 0.1 (strong), 0.01 to 0.03 (very strong), and <0.01 (extreme). BF > 1 provides evidence toward the null hypothesis: 1 to 3 (anecdotal), 3 to 10 (moderate), 10 to 30 (strong), 30 to 100 (very strong), and >100 (extreme).

To further describe and visualize the relationship between cognitive performance and psychopathology, all participants were divided into deciles (*n* = 648 in each bin) based on their *z* score from the cognitive normative model, with low bins indicating good cognitive performance. Next, within each bin, we computed the number and proportion of clinical risk group participants as well as the corresponding odds ratio (OR), based on varying clinical thresholds of 1, 1.5, 2, and 3 SD above the mean for the clinical ICs. OR was calculated as

$$OR=\frac{\left(n\right)\;exposed\;cases\;\times \;\left(n\right)\;unexposed\;noncases}{\left(n\right)\;exposed\;noncases\;\times \;\left(n\right)\;unexposed\;cases}$$

where exposed are participants with a psychopathological symptom score higher than 2 SD from the mean, cases are the participants placed in the lowest bins (i.e., poorest performance), and noncases have the best cognitive performance.

## Results

### Association Between Normative Model of Cognition and Psychopathology Scores

**Figure 3:**
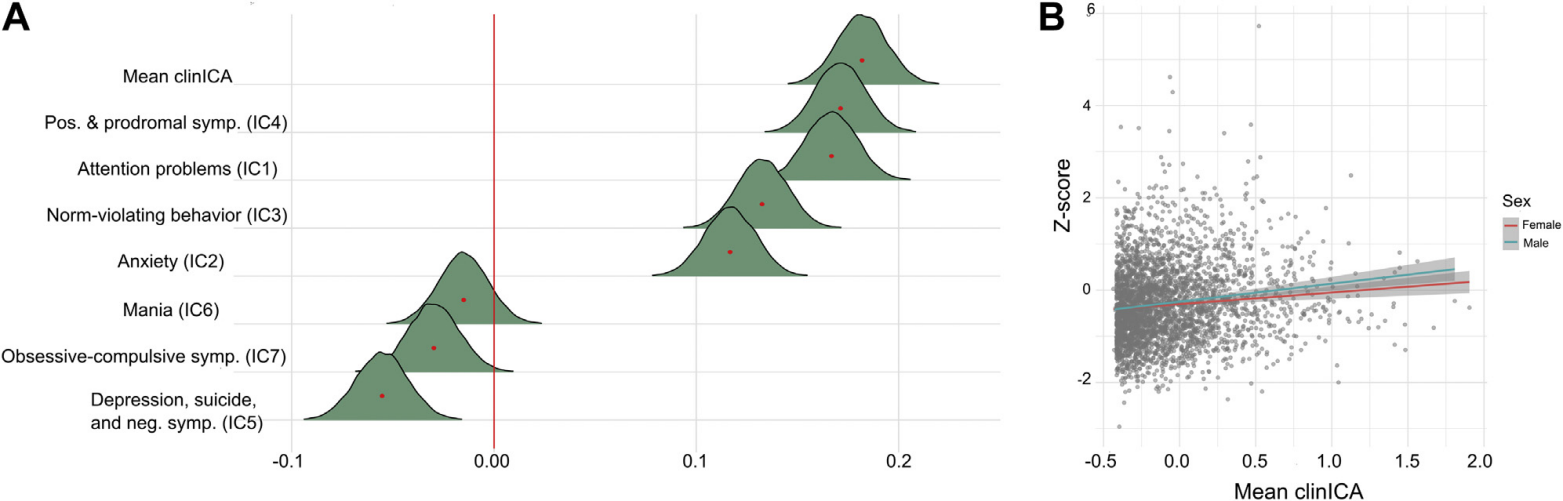
Associations between cognition and psychopathology. **(A)** Posterior distributions for the association between the different psychopathology measures and the cognitive deviation score. The mean estimate for each association is indicated with a red dot. The colored area represents the uncertainty of the mean, measured by the 95% credible interval of the posterior distribution. **(B)** Scatterplot displaying the relationship between *z* score of cognitive performance and general psychopathology. IC, independent component; ICA, independent component analysis; neg. symp., negative symptoms.

Figure 3 shows the posterior distributions for the association between the different psychopathological domain scores and the cognitive deviation score. The tests confirmed extreme evidence in favor of an association between cognitive deviation score and the general psychopathology factor (BF ≤ 0.001, β = 0.182), and the clinical factors reflecting positive and prodromal psychosis symptoms (BF ≤ 0.001, β = 0.171), attention problems (BF ≤ 0.001, β = 0.167), norm-violating behavior (BF ≤ 0.001, β = 0.133), and anxiety (BF ≤ 0.001, β = 0.117), indicating more negative deviations from the cognitive norm (i.e., poorer performance) with more severe psychopathological symptoms (see Table S4 for summary statistics). The tests also revealed extreme evidence in favor of a negative association between cognitive deviation scores and the component reflecting depression, suicide, and negative symptoms (BF = 0.002, β = −0.055), indicating better cognitive performance with higher burden. For the remaining psychopathological domain components, the models revealed strong and anecdotal evidence in favor of no associations for symptoms of mania (BF = 19.37, β = −0.015) and obsessive-compulsive disorder (BF = 2.654, β = −0.030), respectively.

**Figure 4:**
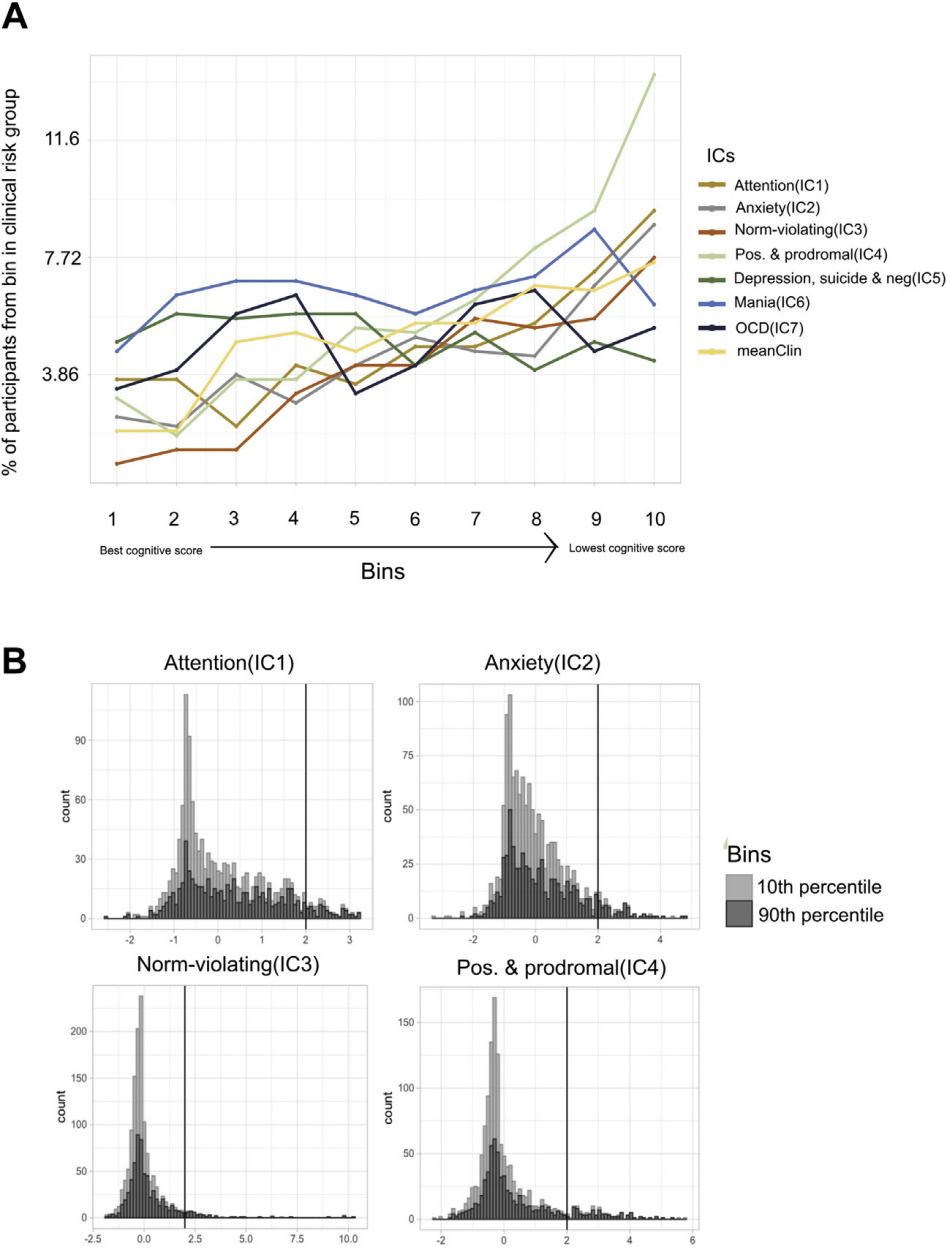
Proportion of high-risk individuals among different groups based on cognitive performance. **(A)** Individuals in the low bins perform better than the average, and individuals in the high bins perform worse than average. The graphs show the proportion of participants in each bin that score over the given clinical cutoff (2 SD from the mean) for each of the psychopathological domains. **(B)** Plots showing the distribution of four of the psychopathological domains for the participants who are placed in the 10th percentile of cognitive performance (light gray) and in the 90th percentile of cognitive performance. IC, independent component; OCD, obsessive-compulsive disorder.

Figure 4 shows the proportion of individuals with a psychopathological domain score >2 SD above the mean within each bin based on the cognitive performance score. Strikingly, for all psychopathological domains except depression, suicide, and negative symptoms (IC5, OR = 0.87); mania (IC6, OR = 1.36); and obsessive-compulsive symptoms (IC7, OR = 1.63), the odds of individuals being in the psychopathology risk group were 2.5 (IC1), 3.81 (IC2), 8.95 (IC3), 4.97 (IC4), and 4.1 (meanClinICA) times higher among the participants in the poorest performance bin than the participants placed in the best performance bin. This indicates that among the participants with poorer cognition, there is a substantially higher number of people who have high scores on general and/or domain-specific psychopathology.

### Associations Between Cognitive Deviance Scores and Polygenic Risk Scores

**Figure 5:**
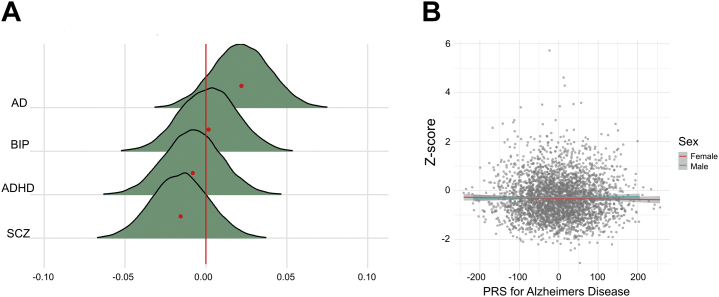
Associations between polygenic risk and cognition. **(A)** Posterior distributions for the associations between polygenic risk scores (PRS) and the cognitive deviation score. Mean estimate is indicated with a red dot. The colored area represents the uncertainty of the mean measured by the 95% credible interval of the posterior distribution. **(B)** Scatterplot displaying the relationship between *z* score on cognitive performance and PRS for Alzheimer’s disease (AD). ADHD, attention-deficit/hyperactivity disorder; BIP, bipolar disorder; SCZ, schizophrenia.

Figure 5 shows the posterior distributions for the association between polygenic risk scores and the cognitive deviation score (see Table S4 for summary statistics). The model revealed strong evidence for no (null) association between cognitive deviance and polygenic scores for schizophrenia (BF = 18.42, β = −0.016), bipolar disorder (BF = 25.90, β = 0.002), AD (BF = 13.10, β = 0.022), and ADHD (BF = 25.03, β = −0.008).

## Discussion

Subtle cognitive deficits are assumed to accompany or even precede the emergence of clinical symptoms in early phases of many mental disorders. The overall aim of this study was to examine if deviations in cognitive performance during childhood and adolescence could serve as an early indicator of mental disorder. To this end, we tested for associations between deviations from a normative trajectory of general cognitive function and both psychopathology domains and polygenic scores reflecting the cumulative genetic load associated with major early- and late-life mental and cognitive disorders. Using normative modeling built on cognitive test performance, we estimated normative cognitive trajectories and tested for associations between individual deviance scores and psychopathology and polygenic scores for schizophrenia, bipolar disorder, ADHD, and AD. While a previous study reported an exclusive association between cognition and psychotic symptoms in the Philadelphia Neurodevelopmental Cohort (44), our analysis also revealed associations with other dimensions of psychopathology. Bayesian statistics revealed strong evidence for associations between cognitive deviation and several domains of psychopathology, including a general psychopathology factor and symptom domains reflecting anxiety, positive psychotic and prodromal symptoms, norm-violating behavior, and attention problems. Here, higher symptom load was associated with more negatively deviating cognitive performance, and OR was an order of magnitude higher among the lowest-performing compared with the highest-performing participants. The four implicated symptom categories have previously been classified as belonging to separate empirical symptom factors often termed internalizing, externalizing, and thought disorder symptoms (45). Thus, our results indicate a generalized association between cognition and psychopathology. In contrast, the mania and obsessive-compulsive disorder symptom domains showed strong and anecdotal evidence for null associations between cognitive performance.

The association between cognitive performance and general psychopathology was expected based on previous research in children and adolescents (28,44,46,47), and similar broad psychopathological patterns have also been found when investigating the association between brain white matter characteristics and psychopathology (28). Beyond the general psychopathology factor, the psychosis positive and prodromal symptoms factor showed the strongest association with cognitive deviation. A possible explanation for psychosis positive and prodromal symptoms having the strongest association is that psychosis symptoms may be considered among the more extreme part of the psychopathology severity spectrum and may thus indicate stronger clinical risk and a more severe outcome. Thus, participants with high scores on the psychosis component would also be expected to show more negative deviations from the norm on cognitive performance. Supporting these findings, a previous neuroimaging study in a partly overlapping sample reported that the psychosis domain converged on regions of the cerebellum that have shown functional connectivity with the parietal cerebral network, which is associated with cognitive control processes (48).

While the majority of the associations reflected lower cognitive performance with higher levels of psychological symptoms, stronger symptoms of depression, suicidal ideation, and negative psychosis symptoms were associated with higher cognitive performance. The questionnaire items loading most heavily on this dimensional factor were “having an altered perception of yourself and/or the world,” “feeling disconnected from yourself or your life,” and “feeling sad or depressed most of the time.” These findings were unexpected given that cognitive dysfunction is considered a pathological feature of major depressive disorder (49). A possible explanation is that this dimensional component did not pick up core depression symptoms but rather captures, for instance, personality traits.

In line with the linear associations, the proportion of participants with extreme symptom scores (>2 SD above the mean) was substantially higher among the lowest cognitively performing participants compared with the highest-performing participants for most measures of psychopathology, with ORs suggesting almost five times higher probability of belonging to the clinical risk group among the lowest-performing decile compared with the highest-performing decile. While follow-up studies are required to assess the predictive value of the cognitive deviation score for future transition to a psychotic state or even a diagnosis of schizophrenia or other psychotic disorders, these results support the intuitive link between cognitive function and clinical signs of incipient or emerging psychopathology in children and adolescents. However, participants with extreme symptom loads were represented across all performance bins, possibly indicating subgroups of high-performing individuals with high clinical risk or psychological distress. Previous normative modeling studies on brain magnetic resonance imaging features have found substantial interindividual heterogeneity in various brain structures among patients with schizophrenia, bipolar disorder, autism spectrum disorder, and ADHD (26,50, 51, 52, 53, 54). While we did not pursue a classical case-control design in this work, our findings indicate that this interindividual heterogeneity also is present in terms of cognitive performance. Beyond the general patterns revealed by the linear models, these individual differences may be highly relevant for clinical decision making.

In general, idiosyncratic patterns of deviations revealed using normative modeling support the intuitive notion that group mean differences often represent inaccurate reflections of the deviations seen at an individual level. Because cognitive deviations in the prodromal phase have been found to be associated with more severe clinical phenotypes later in life (55), accurate modeling of cognitive maturity and deviation using normative modeling has the potential to provide valuable information for early detection of at-risk youth. This work provides a step toward the aim of adapting the flexible framework of normative modeling to map the individualistic patterns of cognition and their relationship to clinical risk. In general, the model apparently adequately detected individuals with a lower performance than expected based on the estimated trajectory. Owing to ceiling effects in the cognitive performance data, the model was likely less accurate in parsing the higher end of the function spectrum. Our normative model showed that the Gaussian process regression performed well out of sample. However, future work might consider alternative algorithms such as Bayesian linear regression with warping (56), which may improve the estimation of the mean and variance further.

Bayesian regression analysis revealed strong evidence for no associations between cognitive deviation and polygenic scores for schizophrenia, bipolar disorder, ADHD, and AD. Previous studies have provided mixed findings. In healthy adults, no significant associations were reported between general cognitive function and polygenic scores for ADHD, bipolar disorder, and AD (57,58). Others have reported associations between polygenic risk score for ADHD and more specific cognitive functions, such as working memory (59). Children, adolescents, and young adults carrying risk alleles for AD have also been shown to exhibit abnormal patterns of brain connectivity including brain regions involved in memory performance and inhibitory control (60,61). Furthermore, studies in healthy adults have reported negative associations between polygenic scores for schizophrenia and IQ (58,62), but others have found no associations (63,64). Studies in children and adolescents have also revealed mixed results, with some reporting positive (36), others negative (65), and some reporting no associations (62) between polygenic scores for schizophrenia and cognitive performance. A possible explanation for the lack of association with polygenic scores can be that the environment plays a vital role in this life period and that the genetic effects accumulate more over the life span.

The results of this study should be interpreted while considering its limitations. Most importantly, the cross-sectional nature of the investigations does not allow us to assess the predictive value of the cognitive deviation scores for future mental disorders. Similarly, both the performance scores and the symptom load reflect a snapshot of the current state of the individual. Future follow-up studies may be able to assess the predictive value for long-term mental health and outcome and assess important aspects of the temporal dynamics of the measured psychological factors. For example, symptom scores may show substantial day-to-day variability (66), and delineating state-like from trait-like characteristics of mental health is highly relevant in a clinical setting. A limitation in the genetic analysis is that we only included a subsample with European ancestry, limiting the generalizability to youth with other ancestries.

In conclusion, individual deviance in cognitive performance is related to general and specific domains of psychopathology in youth, with a substantially higher proportion of individuals with extreme symptom load among the lowest-performing participants compared with the highest-performing participants. While follow-up studies are needed to assess the predictive value for future development of mental illness, these results support the close links between emerging psychopathology and cognitive function in youth.

## Supplementary Material

Supplementary Material

Key Resources Table
