# Supplementary material for: Mapping Normative Trajectories of Cognitive Function and Its Relation to Psychopathology Symptoms and Genetic Risk in Youth
Source: Biol Psychiatry Glob Open Sci. 2022 Feb 1;3(2):255–63. doi: 10.1016/j.bpsgos.2022.01.007 (PMC10140446; doi:10.1016/j.bpsgos.2022.01.007)
Supplement: Supplementary Material [file mmc1.pdf]

Mapping Normative Trajectories of Cognitive Function and Its Relation to Psychopathology Symptoms and Genetic Risk in Youth

Supplemental Information

Supplemental table S1. Overview of the demographics and cognitive scores of the main sample and the subsample. M = mean, sd = standard deviations.

	**Main sample (N=6481)**	**Subsample (n=3175)**
**Age**	8-21 [M = 13.6, sd = 3.6]	8-21 [M=13.6, sd=3.6]
**Sex**	3377 girls (52%)	1581 girls (50%)
**Ethnicity (self-report)**	3476 European American (53,6%)<br>2267 African American (35%)<br>3 Native American (0.004%)<br>614 Mixed (9.5 %)<br>121 Other (1.9 %)	3175 European American
**Wide Range Assessment Test**	M= 50.5, sd = 10.0	M = 53.3, sd = 9.0
**The Penn Age Differentiation Test**	M =69.2, sd = 12.1	M = 69.7, sd = 11.9
**The Penn Face Memory Test**	M = 30.4, sd = 4.3	M = 30.9, sd = 4.2
**Penn Emotion Identification Test**	M = 33.2, sd = 3.4	M = 33.3, sd = 3.2
**Penn Word Memory Test**	M = 36.8, sd = 3.4	M = 37.0, sd = 3.0
**Penn Verbal Reasoning Test**	M = 10.7, sd = 3.1	M = 11.6, sd = 2.8
**Penn Emotion Differentiation Test**	M = 72.0, sd = 11.9	M = 73.5, sd = 10.9
**Penn Motor Praxistask**	M = 730.1, sd = 140.7	M = 722.4, sd = 132.3
**Penn Matrix Reasoning Test**	M = 49.9, sd = 19.5	M = 53.8, sd = 19.2
**Finger Tapping Test**	M = 95.7, sd = 14.2	M = 96.2, sd = 14.2
**Visual Object Learning Test**	M = 15.6, sd = 2.5	M = 15.9, sd = 2.3
**Letter N-Back Test**	M = 17.5, sd = 2.8	M = 17.9, sd = 2.8
**Penn Conditional Exclusion Test (category)**	M = 2.6, sd = 0.8	M = 2.7, sd = 0.7
**Penn Conditional Exclusion Test (accuracy)**	M = 14499.7, sd = 10471	M = 15539.3, sd = 10524
**Penn Continuous Performance Test**	M = 50.8, sd = 8.6	M = 51.7, sd = 7.8
**Penn Continuous Performance Test, RT**	M = 494.8, sd = 68.8	M = 495.1, sd = 70.0
**Penn Line Orientation Test**	M = 37.8, sd = 18.5	M = 42.6, sd = 18.1

Supplemental table S2. All included cognitive test measures and their contributions to the first five principal components from the Principal Component Analysis.

Cognitive Tests	PC1	PC2	PC3	PC4	PC5
The Penn Age Differentiation Test	7.613	0.111	0.905	0.061	17.390
The Penn Face Memory Test	6.030	2.126	7.346	9.950	0.003
Penn Emotion Identification Test	3.308	0.074	0.224	28.223	22.656
Penn Word Memory Test	2.918	5.965	10.061	14.069	8.969
Penn Verbal Reasoning Tes	10.430	0.032	0.111	4.546	0.041
Penn Emotion Differentiation Test	8.405	0.023	0.739	0.255	12.552
Penn Motor Praxistask	2.845	10.523	3.800	11.076	16.399
Penn Matrix Reasoning Test	8.387	0.299	0.548	11.476	0.080
Finger Tapping Test	6.546	12.935	2.298	0.728	1.530
Visual Object Learning Test	2.679	12.113	13.021	0.122	14.686
Letter N-Back Test	6.291	0.121	0.013	2.923	1.984
Penn Conditional Exclusion Test	3.910	18.051	21.772	0.562	0.028
Penn Conditional Exclusion Test	2.094	18.306	34.177	0.464	0.011
Penn Continuous Performance Test	4.865	2.109	0.106	0.617	0.617
Penn Continuous Performance Test	4.948	16.027	4.118	1.619	2.519
Penn Line Orientation Test	7.809	0.009	0.764	11.421	0.271
Wide Range Assessment Test(Reading/IQ)	10.923	1.177	0.003	1.888	0.265

Supplemental table S3. Overview of all clinical items included in independent component analysis

dbGaP Study Accession	Variable name	Item question
phv00194007.v1.p1	INT_TYPE	AP=Adult Proband (ages 18 and up); MP= Middle Proband (ages 11-17); MI=Middle Informant; YPI= Young Proband Informant (for ages 8-10)
		**Attention Deficit Disorder (ADD)**
phv00194008.v1.p1	ADD011	Did you often have trouble paying attention or keeping your mind on your school, work, chores, or other activities that you were doing?
phv00194009.v1.p1	ADD012	Did you often have problems following instructions and often fail to finish school, work, or other things you meant to get done?
phv00194010.v1.p1	ADD013	Did you often dislike, avoid, or put off school or homework (or any other activity requiring concentration)
phv00194011.v1.p1	ADD014	Did you often lose things you needed for school or projects at home (assignments or books) or make careless mistakes in school work or other activities?
phv00194012.v1.p1	ADD015	Did you often have trouble making plans, doing things that had to be done in a certain kind of order, or that had a lot of different steps?
phv00194013.v1.p1	ADD016	Did you often have people tell you that you did not seem to be listening when they spoke to you or that you were daydreaming?
phv00194017.v1.p1	ADD020	Did you often have difficulty sitting still for more than a few minutes at a time, even after being asked to stay seated, or did you often fidget with your hands or feet or wiggle in your seat or were you "always on the go"?
phv00194018.v1.p1	ADD021	Did you often blurt out answers to other people's questions before they finished speaking or interrupt people abruptly?
phv00194019.v1.p1	ADD022	Did you often join other people's conversations or have trouble waiting your turn (e.g., waiting in line, waiting for a teacher to call on you in class)?
		**Agoraphobia (AGR)**
phv00194035.v1.p1	AGR001	Have you ever been very nervous or afraid of: being in crowds (for example, a classroom, cafeteria, restaurant, or movie theater)?
phv00194036.v1.p1	AGR002	Have you ever been very nervous or afraid of: going to public places (such as a store or shopping mall)?
phv00194037.v1.p1	AGR003	Have you ever been very nervous or afraid of: being in an open field?
phv00194038.v1.p1	AGR004	Have you ever been very nervous or afraid of: going over bridges or through tunnels?
phv00194039.v1.p1	AGR005	Have you ever been very nervous or afraid of: traveling by yourself?
phv00194040.v1.p1	AGR006	Have you ever been very nervous or afraid of: traveling away from home?
phv00194041.v1.p1	AGR007	Have you ever been very nervous or afraid of: traveling in a car?
phv00194042.v1.p1	AGR008	Have you ever been very nervous or afraid of: using public transportation like a bus or SEPTA?
		**Conduct disorder (CDD)**
phv00194062.v1.p1	CDD001	Was there ever a time when you often did things that got you into trouble with adults like lying or stealing (something worth more than $5, from family, others, or stores)?
phv00194064.v1.p1	CDD002	Did you ever skip school, stay out at night later than you were supposed to (more than 2 hours), or run away from home overnight?
phv00194066.v1.p1	CDD003	Did you ever set fires, break into cars, or destroy someone else's property on purpose?

phv00194067.v1.p1	CDD004	Do you have a probation officer or have you ever been on probation?
phv00194068.v1.p1	CDD005	Did you often bully others (hitting, threatening or scaring someone who was younger or smaller), threaten or frighten someone on purpose, or often start physical fights with others?
phv00194069.v1.p1	CDD006	Have you ever been physically cruel to an animal or person (on purpose)?
phv00194070.v1.p1	CDD007	Did you ever: try to hurt someone with a weapon (a bat, brick, broken bottle, knife, or gun)?
phv00194071.v1.p1	CDD008	Did you ever: threaten someone?
phv00194072.v1.p1	CDD009	Conduct Disorder: Did you ever: hold someone up?
phv00194073.v1.p1	CDD010	Conduct Disorder: Did you ever: attack someone to steal from them?
phv00194074.v1.p1	CDD011	Did you ever: trick or threaten someone into having sex with you, or did anyone ever accuse you of making them do something sexual?
		**Depression (DEP)**
phv00194091.v1.p1	DEP001	Has there ever been a time when you felt sad or depressed most of the time?
phv00194092.v1.p1	DEP002	Has there ever been a time when you cried a lot, or felt like crying?
phv00194094.v1.p1	DEP004	Has there ever been a time when you felt grouchy, irritable or in a bad mood most of the time; even little things would make you mad?
phv00194096.v1.p1	DEP006	Has there ever been a time when nothing was fun for you and you just weren't interested in anything?
		**Eating disorder (EAT)**
phv00194133.v1.p1	EAT001	Was there ever a time when you felt really fat or heavy, but other people said that you were too thin?
phv00194139.v1.p1	EAT007	Has there been a time when your eating was out of control - you'd eat a large amount of food in a short period of time and could not stop yourself?
		**Generalized anxiety (GAD)**
phv00194149.v1.p1	GAD001	Have you ever been a worrier?
phv00194150.v1.p1	GAD002	Did you worry a lot more than most children/people your age?
		**Mania/Hypomania (MAN)**
phv00194185.v1.p1	MAN001	Have there been times when you were much more active, excited or energetic than usual, had problems sitting still, or needed to move around a lot?
phv00194186.v1.p1	MAN002	Has there ever been a time when you felt so full of energy that you couldn't stop doing things and didn't get tired?
phv00194187.v1.p1	MAN003	Has there ever been a time when you felt like you hardly needed sleep?
phv00194188.v1.p1	MAN004	Have there been times when you kept talking a lot, couldn't stop talking, talked faster than usual, had thoughts faster than usual, or had so many ideas in your head that you could hardly keep track of them?
phv00194189.v1.p1	MAN005	Have you ever had a time when you felt much more happy or excited than you usually do when there was nothing special going on?
phv00194190.v1.p1	MAN006	Have you ever had a time when you felt like you could do almost anything?
phv00194191.v1.p1	MAN007	Has there ever been a time when you felt unusually grouchy, cranky, or irritable; when the smallest things would make you really mad?

		**Obsessive compulsive disorder (OCD)**
phv00194390.v1.p1	OCD001	Have you ever been bothered by thoughts that don't make sense to you, that come over and over again and won't go away, such as concern with harming others/self?
phv00194391.v1.p1	OCD002	Have you ever been bothered by thoughts that don't make sense to you, that come over and over again and won't go away, such as pictures of violent things?
phv00194392.v1.p1	OCD003	Have you ever been bothered by thoughts that don't make sense to you, that come over and over again and won't go away, such as thoughts about contamination/germs/illness?
phv00194393.v1.p1	OCD004	Have you ever been bothered by thoughts that don't make sense to you, that come over and over again and won't go away, such as fear that you would do something/say something bad without intending to?
phv00194394.v1.p1	OCD005	Have you ever been bothered by thoughts that don't make sense to you, that come over and over again and won't go away, such as feelings that bad things that happened were your fault?
phv00194395.v1.p1	OCD006	Have you ever been bothered by thoughts that don't make sense to you, that come over and over again and won't go away, such as forbidden/bad thoughts?
phv00194396.v1.p1	OCD007	Have you ever been bothered by thoughts that don't make sense to you, that come over and over again and won't go away, such as need for symmetry/exactness?
phv00194397.v1.p1	OCD008	Have you ever been bothered by thoughts that don't make sense to you, that come over and over again and won't go away, such as religious thoughts?
phv00194400.v1.p1	OCD011	Have you ever had to do something over and over again - that would have made you feel really nervous if you couldn't do it, like: cleaning or washing (for example, your hands, house)?
phv00194401.v1.p1	OCD012	Have you ever had to do something over and over again - that would have made you feel really nervous if you couldn't do it, like: counting?
phv00194402.v1.p1	OCD013	Have you ever had to do something over and over again - that would have made you feel really nervous if you couldn't do it, like: checking (for example, doors, locks, ovens)?
phv00194403.v1.p1	OCD014	Have you ever had to do something over and over again - that would have made you feel really nervous if you couldn't do it, like: getting dressed over and over again?
phv00194404.v1.p1	OCD015	Have you ever had to do something over and over again - that would have made you feel really nervous if you couldn't do it, like: going in and out a door over and over again?
phv00194405.v1.p1	OCD016	Have you ever had to do something over and over again - that would have made you feel really nervous if you couldn't do it, like: ordering or arranging things?
phv00194406.v1.p1	OCD017	Have you ever had to do something over and over again - that would have made you feel really nervous if you couldn't do it, like: doing things over and over again at bedtime, like arranging the pillows, sheets, or other things?
phv00194407.v1.p1	OCD018	Have you ever saved up so many things that people complained or they got in the way
phv00194408.v1.p1	OCD019	Do you feel the need to do things just right (like they have to be perfect)?
		**Oppositional defiant disorder (ODD)**
phv00194431.v1.p1	ODD001	Was there a time when you often did things that got you into trouble with adults such as losing your temper, arguing with or talking back to adults, or being grouchy or irritable with them?
phv00194432.v1.p1	ODD002	Was there a time when you often got into trouble with adults for refusing to do what they told you to do or for breaking rules at home/school?
phv00194434.v1.p1	ODD003	Did you often annoy other people on purpose or blame other people for your mistakes (excluding siblings)?
phv00194436.v1.p1	ODD005	Did you ever get into trouble for getting even with other people by doing things to hurt them, telling lies about them, or messing up their things?
phv00194437.v1.p1	ODD006	Were you often irritable or grouchy, or did you often get angry because you thought that things were unfair?
		**Panic disorder (PAN)**
phv00194448.v1.p1	PAN001	Have you ever had an attack like this?

phv00194449.v1.p1	PAN003	Has there ever been a time when all of a sudden you felt very, very scared or uncomfortable - and your chest hurt, you couldn't catch your breath, your heart beat very fast, you felt very shaky, and sweaty/tingly/numb in your hands or feet?
phv00194450.v1.p1	PAN004	Has there ever been a time when all of a sudden, you felt that you were losing control, something terrible was going to happen, that you were going crazy, or going to die?
		**Specific phobia (PHB)**
phv00194468.v1.p1	PHB001	have you ever been very nervous or afraid of animals or bugs, like dogs, snakes, or spiders?
phv00194469.v1.p1	PHB002	have you ever been very nervous or afraid of being in really high places, like a roof or tall building?
phv00194470.v1.p1	PHB003	have you ever been very nervous or afraid of water or situations involving water, such as a swimming pool, lake, or ocean?
phv00194471.v1.p1	PHB004	have you ever been very nervous or afraid of storms, thunder, or lightning?
phv00194472.v1.p1	PHB005	have you ever been very nervous or afraid of doctors, needles, or blood?
phv00194473.v1.p1	PHB006	have you ever been very nervous or afraid of closed spaces, like elevators or closets?
phv00194474.v1.p1	PHB007	have you ever been very nervous or afraid of flying or airplanes?
phv00194475.v1.p1	PHB008	have you ever been very nervous or afraid of any other things or situations?
		**Psychosis (PSY)**
phv00194490.v1.p1	PSY001	Have you ever heard voices when no one was there?
phv00194507.v1.p1	PSY020	Has there ever been anything unusual about the way things smelled or felt or looked?
phv00194518.v1.p1	PSY029	Have you ever seen visions or seen things which other people could not see?
phv00194531.v1.p1	PSY050	Have you ever smelled strange odors other people could not smell?
phv00194539.v1.p1	PSY060	Have you ever had strange feelings in your body like things were crawling on you or someone touching you and nothing or no one was there?
phv00194546.v1.p1	PSY071	Have you ever believed in things and later found out they weren't true, like people being out to get you, or talking about you behind your back, or controlling what you do or think?
		**Post-traumatic stress (PTD)**
phv00194609.v1.p1	PTD001	Have you ever been in a flood or a tornado or an earthquake or a hurricane or some other natural disaster where you thought you were going to die or be seriously hurt?
phv00194610.v1.p1	PTD002	Have you ever been in a situation where you thought you or someone close to you was going to be killed or be hurt very badly (e.g. family violence)?
phv00194611.v1.p1	PTD003	Have you ever been attacked by somebody or badly beaten?
phv00194612.v1.p1	PTD004	Have you ever been very upset by someone forcing you to do something sexual?
phv00194614.v1.p1	PTD006	Have you ever been threatened with a weapon?
phv00194615.v1.p1	PTD007	Have you ever been in a bad accident?
phv00194616.v1.p1	PTD008	Other than television or at the movies, have you ever seen or heard somebody get killed or get hurt very badly or die?
phv00194617.v1.p1	PTD009	Have you ever been very upset by seeing a dead body or by seeing pictures of the dead body of somebody you knew well?

		**General probes (SCR)**
phv00194639.v1.p1	SCR001	Have you ever talked to a counselor, psychologist, social worker, psychiatrist or some other professional about your feelings or problems with your mood or behaviors?
phv00194644.v1.p1	SCR006	Are you currently taking medication because of your emotions and/or behaviors?
phv00194645.v1.p1	SCR007	Have you ever had to go to a hospital and stay overnight because of problems with your mood, feelings, or how you were acting?
		**Separation anxiety (SEP)**
phv00194654.v1.p1	SEP500	Since you were 5 years old, has there ever been a time when you had a lot of worries about your (attachment figures) and were very upset or got sick (for example, felt sick to your stomach, headaches, thrown-up) when you were away from him/her?
phv00194656.v1.p1	SEP508	Has there ever been a time when you wanted to stay home from school or not go to other places (for example, sleep-overs) without your (attachment figures)?
phv00194657.v1.p1	SEP509	When you knew that you were going to be away from home or (attachment figure(s)), did you get very upset and worry (e.g., when you learned (attachment figure(s)) were going on an upcoming trip or night out)?
phv00194658.v1.p1	SEP510	Did you ever worry/have bad dreams about something terrible happening to you or your (attachment figures) so that you would not see them again?
phv00194659.v1.p1	SEP511	Were you scared to be alone in your room (or any place in your house) or did you need your (attachment figure(s)) to stay with you while you fell asleep?
		**Structural interview for prodromal symptoms (SIP)**
phv00194672.v1.p1	SIP001	TROUBLE WITH FOCUS AND ATTENTION Severity Scale
phv00194674.v1.p1	SIP003	I think that I have felt that there are odd or unusual things going on that I can't explain.
phv00194675.v1.p1	SIP004	I think that I might be able to predict the future.
phv00194676.v1.p1	SIP005	I may have felt that there could possibly be something interrupting or controlling my thoughts, feelings, or actions.
phv00194677.v1.p1	SIP006	I have had the experience of doing something differently because of my superstitions.
phv00194678.v1.p1	SIP007	I think I may get confused at times whether something I experience or perceive may be real or may be just part of my imagination or dreams.
phv00194679.v1.p1	SIP008	I have thought that it might be possible that other people can read my mind, or that I can read others' minds
phv00194680.v1.p1	SIP009	I wonder if people may be planning to hurt me or even may be about to hurt me.
phv00194681.v1.p1	SIP010	I believe that I have special natural or supernatural gifts beyond my talents and natural strengths.
phv00194682.v1.p1	SIP011	I think I might feel like my mind is "playing tricks" on me.
phv00194683.v1.p1	SIP012	I have had the experience of hearing faint or clear sounds of people or a person mumbling or talking when there is no one near me.
phv00194684.v1.p1	SIP013	I think that I may hear my own thoughts being said out loud.
phv00194685.v1.p1	SIP014	I have been concerned that I might be "going crazy."
phv00194698.v1.p1	SIP027	Do people ever tell you that they can't understand you?
phv00194699.v1.p1	SIP028	Do people ever seem to have difficulty understanding you?
phv00194700.v1.p1	SIP030	Changes in speech, disorganized communication, tangential speech Severity Scale

phv00194702.v1.p1	SIP032	Do you ever feel a loss of sense of self or feel disconnected from yourself or your life?
phv00194703.v1.p1	SIP033	Has anyone pointed out to you that you are less emotional or connected to people than you used to be?
phv00194704.v1.p1	SIP035	Changes in perception of self, others, or the world in general: Severity Scale
phv00194706.v1.p1	SIP037	EXPRESSION OF EMOTION: Severity Scale
phv00194707.v1.p1	SIP038	Within the past 6 months, are you having a harder time getting your work or schoolwork done?
phv00194708.v1.p1	SIP039	Within the past 6 months, are you having a harder time getting normal activities done?
phv00194709.v1.p1	SIP041	Occupational Functioning Severity Scale
phv00194711.v1.p1	SIP043	Avolition Severity Scale
		**Social Anxiety (SOC)**
phv00194713.v1.p1	SOC001	was there ever a time in your life when you felt afraid or uncomfortable or really, really shy with people, like meeting new people, going to parties, or eating or drinking, writing or doing homework in front of others?
phv00194714.v1.p1	SOC002	was there ever a time in your life when you felt afraid or uncomfortable talking on the telephone or with people your own age who you don't know very well?
phv00194715.v1.p1	SOC003	was there ever a time in your life when you felt afraid or uncomfortable when you had to do something in front of a group of people, like speaking in class?
phv00194716.v1.p1	SOC004	was there ever a time in your life when you felt afraid or uncomfortable acting, performing, giving a talk/speech, playing a sport or doing a musical performance, or taking an important test or exam (even though you studied enough)?
phv00194717.v1.p1	SOC005	was there ever a time in your life when you felt afraid or uncomfortable because you were the center of attention and were concerned something embarrassing might happen and you felt very afraid or felt uncomfortable?
		**Depression (DEP)**
phv00194734.v1.p1	SUI001	Have you ever thought a lot about death or dying?
phv00194735.v1.p1	SUI002	Have you ever thought about killing yourself?

Supplemental figure S1. Distributions of the psychopathological domain scores.

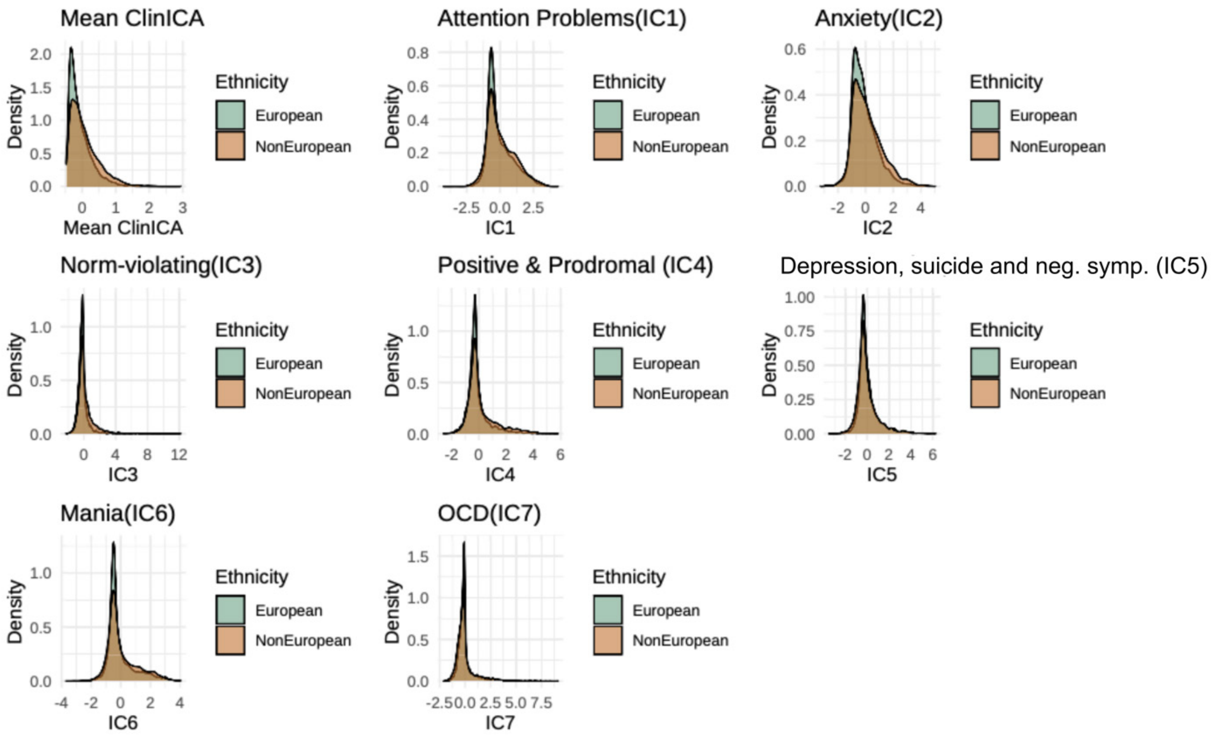

Supplemental table S4. Summary stats for Bayesian statistical analysis

y	prs/ic	estimate	lower95	upper95	p_higher_0	p_lower_0	evidence_0	max_rhat
z-score	AD	0.021950080	-0.01197407	0.05763415	0.8896875	0.1103125	13.10426	1.001039
z-score	ADHD	-0.008150528	-0.04136381	0.02817240	0.3241875	0.6758125	25.03387	1.000244
z-score	SCZ	-0.015641150	-0.04964779	0.01914061	0.1888125	0.8111875	18.42012	1.000920
z-score	BIP	0.001650051	-0.03216607	0.03646846	0.5390625	0.4609375	25.89809	1.000803
z-score	clinIC1	0.16680138	0.14189998	0.191445021	1.000000	0.00000	-2.249087e-21	1.000205
z-score	clinIC2	0.11672618	0.09256281	0.141451023	1.000000	0.00000	-4.4510790e-18	1.000142
z-score	clinIC3	0.13254419	0.10823510	0.156928870	1.000000	0.00000	-7.210982e-18	1.000323
z-score	clinIC4	0.17131404	0.14726898	0.195135735	1.000000	0.00000	9.058838e-17	1.000259
z-score	clinIC5	-0.05528124	-0.08013926	-0.029901293	0.000000	1.00000	2.025229e-03	1.000294
z-score	clinIC6	-0.01498402	-0.04032307	0.008449277	0.1124375	0.8875625	1.937020e+01	1.000304
z-score	clinIC7	-0.02973615	-0.05423553	-0.004455241	0.0095625	0.9904375	2.654557e+00	1.000122
z-score	MeanClin	0.18199089	0.15811665	0.206694226	1.000000	0.00000	8.618264e-16	1.000373
